# Reliability and validity of the Amharic version of European Organization for Research and Treatment of cervical Cancer module for the assessment of health related quality of life in women with cervical cancer in Addis Ababa, Ethiopia

**DOI:** 10.1186/s12955-019-1089-x

**Published:** 2019-01-14

**Authors:** Liya Teklu Araya, Gebremedhin Beedemariam Gebretekle, Girma Tekle Gebremariam, Teferi Gedif Fenta

**Affiliations:** 1 Social and Administrative Pharmacy Unit, Department of Pharmaceutics and Social Pharmacy,School of Pharmacy, College of Health Sciences, Addis Ababa University, P.O. Box: 1176, Addis Ababa, Ethiopia; 2 Department of Pharmacology and Clinical Pharmacy, School of Pharmacy, College of Health Sciences, Addis Ababa University, Addis Ababa, Ethiopia

**Keywords:** Cervical Cancer, HRQoL, Validation, Amharic version, EORTC QLQ-CX24

## Abstract

**Background:**

Cervical cancer is among the leading gynecological cancers affecting women worldwide. Maintenance and improvement of cervical cancer patients‘health related quality of life (HRQoL) is an important issue. The cervical cancer specific quality of life module of the European Organization for Research and Treatment of Cancer (EORTC QLQ-CX24) is the most commonly used tool, however, it is not validated in Ethiopia. Hence, the present study aimed to assess the psychometric properties of the tool among Ethiopian cervical cancer patients.

**Methods:**

Hospital based cross-sectional study was done in Tikur Anbessa Specialized Hospital (TASH), Addis Ababa, Ethiopia from January to February, 2018. The module was translated through forward-backward translation approach and pilot tested according to the EORTC Guidelines. One hundered and seventy one patients with confirmed cervical cancer were enrolled for the study. Amharic versions of EORTC QLQ-C30 and EORTC QLQ-CX24 were used to collect data along with socio-demographic and clinical characteristics. Descriptive statistics were used to assess socio-demographic and clinical characteristics of patients. The Psychometric properties of the EORTC QLQ-CX24 were evaluated in terms of acceptability, internal consistency, construct, concurrent and known group validity using SPSS version 22.

**Results:**

One hundred seventy one cervical cancer patients were enrolled in the study, with a mean age of 52.15 ± 10.4 years. The EORTC QLQ-CX24 was found to be acceptable with high compliance and low missing responses. The Cronbach’s alpha ranged from 0.70–0.84, indicating the reliability of the scales. Convergent and discriminant validity in multitrait scaling analysis was adequate. The EORTC QLQ-C30 subscales and EORTC QLQ-CX24 subscales had a weak to strong correlation, indicating concurrent validity. The scales and single-item measures were able to discriminate between subgroups of patients differing with regard to performance status, cancer stage and treatment status, indicating clinical validity.

**Conclusion:**

Amharic version of the EORTC QLQ-CX24 questionnaire is a valid and reliable tool and could be used for clinical and epidemiological cancer researches to study the HRQoL of patients with cervical cancer in Ethiopia.

## Background

Cervical cancer is the fourth most common cancer in women, and the seventh overall, with an estimated 528,000 new cases diagnosed every year. There were 266,000 deaths from cervical cancer worldwide, accounting for 7.5% of all female cancer deaths. An estimated 85% of the globally diagnosed cases and 88% of the deaths due to cervical cancer occur in developing nations [1]. Women in developing nations are at a 35% greater lifetime risk of developing cervical cancer than women in high-income countries [2]; this is due to lack of early detection, screening and diagnosis of the disease at an advanced stage. Cervical cancer is most common in women older than 50 years. However, in developing nations, it is becoming increasingly prevalent among women during their reproductive age of 15–49 years [3]. In Ethiopia, cervical cancer is the second most prevalent cancer among women between 15 and 44 years of age. Every year, about 4648 new cases and 3235 deaths are reported [5]. Thus, maintenance and improvement of cervical cancer patients’ health related quality of life (HRQoL) is becoming an important issue for health care providers in addition to tumor control and increasing survival rate of the patients [5].

HRQoL is a patient-reported outcome that is usually measured using carefully designed and validated instruments [5]. Although HRQoL of cancer patients has been well researched in developed countries, little is known about cancer patients in African countries, facing unique challenges such as poverty, access to health care and under resourced healthcare systems [6]. The European Organization for Research and Treatment of Cancer Quality of Life questionnaire (EORTC QLQ-C30) is a widely used instrument for assessing HRQoL of cancer patients [7]. Amharic is the official language of Ethiopia, spoken by most of the community and the Amharic version of the EORTC QLQ-C30 core questionnaire has been validated to assess HRQoL in Ethiopian cancer patients [8]. The English version of the EORTC QLQ-CX24 cervical cancer module is also available to measure quality of life in cervical cancer patients [9]. The psychometric properties of the EORTC QLQ-CX24 disease specific questionnaire was tested and established in many countries [9–11]. However, there is no cervical cancer specific assessment tool to measure quality of life with cervical cancer patients in Ethiopia. The diversity of culture between the western countries and Ethiopia could result in different interpretations of quality of life, so it is important to assess the acceptability, reliability and validity of the EORTC QLQ-CX24 as well as suitability for Ethiopian cervical cancer patients. Thus, the aim of the study was to assess the acceptability, reliability and validity of the cervical cancer disease specific EORTC QLQ-CX24 module.

## Methods

### Design and participants

A hospital based cross-sectional study was conducted among cervical cancer patients in Tikur Anebessa Specialized Hospital (TASH), Addis Ababa, Ethiopia from January to February, 2018. TASH is the sole oncology referral and radiotherapy center in the entire country. A total of 200 cervical cancer patients were requested for their participations. Participants were considered eligible if they had a pathologically diagnosed cervical cancer, were 18 years and older with the ability to understand and answer survey questions, and were able to speak and communicate well in the local (Amharic) language. Patients were excluded if they were unwilling to participate, unable to understand or complete the interview questionnaires or had a cognitive or mental problem.

### Instruments

#### EORTC QLQ-C30

The 30-items EORTC QLQ-C30 is psychometrically robust, cross-culturally accepted and most frequently used tool to assess HRQoL in cancer patients. It is sub-grouped into 15 domains, including five functional subscales (physical functioning, role functioning, emotional functioning, cognitive functioning, and social functioning); three multi-item symptom subscales (fatigue, nausea/vomiting, and pain); global health status/QoL subscale; and six single items addressing various symptoms and perceived financial impact [7].

#### EORTC QLQ-CX24

The 24-items are classified into three multi-item scales, eleven items with symptom experience domain, three items with body image domain, and four items with sexual/ vaginal functioning domain. The other domains of the questionnaire are single-item scales, including lymphedema, peripheral neuropathy, menopausal symptom, sexual worry, sexual activity, and sexual enjoyment [9].

The standard scoring algorithm recommended by the EORTC was used to linearly transform all scales and item scores to a 0–100 scale; a high score for a functional scale represents a high/healthy level of functioning, whereas a high score for a symptom scale or item represents a high level of symptom or difficulties [12]. The EORTC QLQ-CX24 was translated by professional language speakers employed by the researchers into Amharic version through forward-backward translation approach according to the EORTC guideline [13]. The forward and backward translations were made by different professionals. Furthermore, it was pilot tested on 20 patients with cervical cancer in order to fix modifications to its wording. Thereafter, the authors assessed the EORTC QLQ-CX24 for its content validity as well its cultural adaptability and comprehensiveness in capturing all dimensions of HRQoL specific to cervical cancer.

### Data collection procedure

Patients were enrolled during their visit at the oncology clinic of TASH and informed verbal consent was obtained from all patients. Data was collected by trained oncology nurses. Paper based Amharic versions of the EORTC QLQ-C30 and EORTC QLQ-CX24 module were read out loud for patients in a private room, as the majority of patients were unable to read and write. The data collectors completed the ECOG-PS and a questionnaire concerning sociodemographic and medical characteristics of the patients from the available medical records. Consent to use the English version of the EORTC QLQ-CX24 was obtained from the EORTC research group.

### Statistical analysis

Descriptive statistics (frequency, percentage, mean with standard deviation) were used to report demographic and clinical characteristics. Psychometric properties of the EORTC QLQ-CX24 were assessed in terms of acceptability, internal consistency, construct validity, concurrent validity and known group validity.

The acceptability of the EORTC QLQ-CX24 was assessed in terms of response rate, percentage of missing data, time needed to complete the questionnaire and details of items considered upsetting, confusing or difficult in the questionnaire to respond. The internal consistency of each domains of the EORTC QLQ-CX24 was assessed using Cronbach’s α coefficient, where α ≥ 0.7 indicated adequate scale reliability of the tool [14].

The scale structure was evaluated by multi-trait scaling analysis [10, 15]. This analysis was based on an evaluation of item-scale correlations for item-convergence and item-discriminance. Evidence of item-convergence was defined as a correlation of (r ≥ 0.40) between an item and its own scale. A lower correlation of an item with scales other than its own in comparison to the correlation with its own scale was evidence for item-discriminance. The convergent and discriminant validity for each scale was evaluated by assessing the Pearson’s correlation coefficient. Scaling errors were defined as cases in which an item correlated “significantly less” with its own scale than with other scales. Known group validity was analyzed by independent t-test. This measured the extent to which the EORTC QLQ-CX24 scores were able to discriminate among the groups of different International Federation of Gynecology and Obstetrics (FIGO) stages [16], ECOG-PS [17], and patients being on treatment or not. Pearson correlation analysis was performed to explore the relationship between scales of the EORTC QLQ-C30 and the EORTC QLQ-CX24, this correlation aimed to assess if and to what extent clinical overlapping existed between the EORTC QLQ-C30 and EORTC QLQ-CX24 scales. All statistical procedures were performed using SPSS version 22 (SPSS, Inc. Chicago, USA). Significance level of *p <* 0.05 was employed for the independent t- test and Pearson correlations.

## Results

### Patient characteristics

From the 200 cervical cancer patients, 171 were willing to participate and completed the questionnaires. All patients responded to both the EORTC QLQ-C30 and EORTC QLQ-CX24 without missing response. Majority 118 (69%) of the patients had no formal education, 95 (56%) of the patients were married and their mean age was 52.15 ± 10.4 years. Although the majority166 (97%) of the patients were diagnosed with FIGO stage II-IV, 145 (85%) of the respondents had a good performance status (ECOG-PS (0–1)). Among the patients, 158 (92.4%) of them were admitted as an outpatient. More than half (51%) of the patients were not receiving cancer treatment while 76 (44%) of them were receiving radiotherapy. All patients had previously taken analgesics and had prescriptions to relieve symptoms while they waited their turn to receive cancer treatment (Table 1).

**Table 1:** Socio-demographic and clinical characteristics of cervical cancer patients (*n* = 171)

Variables	Number of patients (%)
Religion	
Orthodox	112 (65.5)
Protestant	35 (20.5)
Muslim	24 (14.0)
Marital Status	
Single	2 (1.2)
Married	96 (56.1)
Divorced	30 (17.5)
Widowed	43 (25.1)
Education	
Unable to read and write	118 (69)
Can read and write	10 (5.8)
Primary education	22 (12.9)
Secondary education	14 (8.2)
FIGO	
Stage 1	6 (3.5)
Stage 2	57 (33.3)
Stage 3	49 (28.7)
Stage 4	59 (34.5)
Patient status	
New Admission	46 (26.9)
Follow up	125 (73.1)
Admission type	
Inpatient	13 (7.6)
Outpatient	158 (92.4)
Current treatment	
Surgery	2 (1.2)
Chemotherapy	5 (2.9)
Radiotherapy	76 (44.4)
Not on treatments	88 (51.5)
ECOG-PS	
0	1 (0.6)
1	144 (84.2)
2	19 (11.1)
3	7 (4.1)

*SD* Standard Deviation, *FIGO* International Federation Gynaecology and Obstetrics, *ECOG-PS* European Cooperative Oncology Group Performance Status; n = Sample size

### Acceptability

The EORTC QLQ-C30 and EORTCQLQ- CX24 were easily understood with full compliance. Of 200 patients, 171 volunteered to take part in the study and respond to the questionnaires without any missing response item. The total time for completion of the interview was approximately 10–15 min, a maximum of 20 min. Most patients 118 (69.0%) reported that all questions are clear and easy to understand, even with uneducated individuals.

### Reliability and validity

Reliability mainly indicates consistency of a measurement tool, ability to measure results in a consistent manner. Meanwhile validity refers to the extent to which a study actually captures or measures what it purports to examine. All of the scales had a Cronbach α ≥ 0.70. Correlation between the items within a scale indicated that all of the items of the EORTC QLQ-CX24 exhibited consistency. In terms of convergent validity, all items showed a good correlation with their own scales (r ≥ 0.4) and were highly correlated with their own scale than the other scales. There were no scaling errors found on the item-discriminance analysis, indicating the divergent validity (Table 2).

**Table 2:** Multi-trait scaling analysis with Pearson correlation between scale items on the EORTC QLQ-CX24

EOTC QLQ-CX24	Item/s number Mean ± SD Cronbach alpha Convergent validity^a^ Discriminant validity^b^				
Symptom scales					
Symptom experience	31–37,39,41–43	42.97 (23.68)	0 .84	0.50–0.78	0.01–0.63
Body image	45–47	45.22 (39.57)	0 .96	0.95–0.97	0.16–0.65
Sexual/Vaginal function	50–53	64.47 (28.84)	0.70	0.62–0.88	0.02–0.79
Symptom items					
Lymphedema	38	12.86 (27.83)	N/A	N/A	0.06–0.33
Peripheral neuropathy	40	40.54 (39.02)	N/A	N/A	0.05–0.49
Menopausal symptoms	44	54.77 (37.50)	N/A	N/A	0.01–0.49
Sexual worry	48	33.52 (46.23)	N/A	N/A	0.10–0.70
Functional Items					
Sexual activity	49	7.49 (22.33)	N/A	N/A	0.20–0.39
Sexual enjoyment	54	38.59 (31.93)	N/A	N/A	0.06–0.39

*SD* standard deviation, *N/A* not applicable; ^a^Item-own scale correlation using Pearson correlation coefficient; ^b^Item-other scale correlation, Pearson correlation coefficient

The correlation of an item and scales of the EORTC QLQ-CX24 with EORTC QLQ-C30 was used to assess the concurrent validity. Correlation between the EORTC QLQ-CX24 and the EORTC QLQ-C30 scales are presented in Table 3. Scales and items of the EORTC QLQ-CX24 were found to be correlated, to some extent, with the scales of the EORTC QLQ-C30. This indicated that the EORTC QLQ-CX24 partially assessed a unique set of HRQoL domains that are currently not covered by the core questionnaire. The symptom experience, body image, peripheral neuropathy and menopausal symptom scales correlated strongly with the EORTC QLQ-C30 functioning scales (r ≥ 0.40). The symptom scales of the EORTC QLQ-CX24 strongly correlated with all subscales of the EORTC QLQ-C30, except for diarrhea and financial impact. The menopausal symptom scale indicated a strong negative correlation with the physical functioning, emotional functioning and social functioning. On the other hand, scales that were related to sexuality (the sexual/vaginal functioning, sexual worry, and sexual enjoyment scales) were weakly correlated with the EORTC QLQ-C30 (r ≤ 0.4).

**Table 3:** Pearson correlation between EORTC QLQ-CX24 and EORTC QLQ-C30

EORTC QLQ-C30	EORTC QLQ-CX24								
	Symptom experience	Body image	Sexual /vaginal functioning	Lymphedema	Peripheral neuropathy	Menopausal symptoms	Sexual worry	Sexual activity	Sexual enjoyment
Functioning scales									
Physical Functioning	−0.56^b^	0.42^b^	0.40	−0.20^b^	− 0.42^b^	− 0.43^b^	− 0.14	− 0.12	− 0.10
Role Functioning	− 0.49^b^	0.42^b^	0.07	− 0.12	− 0.40^b^	− 0.35^b^	− 0.15	− 0.11	− 0.08
Emotional Functioning	− 0.45^b^	0.49^b^	0.31	− 0.14	−0.31^b^	− 0.40^b^	−0.14	− 0.23^b^	0.03
Cognitive Functioning	−0.33^b^	0.35^b^	0.17	−0.29^b^	−0.25^b^	− 0.20^b^	−0.15	− 0.11	−0.08
Social Functioning	−0.48^b^	0.46^b^	0.43	−0.11	−0.39^b^	− 0.43^b^	−0.08	− 0.01	−0.19
Symptom scales									
Fatigue	0.58^b^	0.36^b^	−0.02	0.14	0.31^b^	0.34^b^	0.09	0.05	0.14
Nausea and Vomiting	0.31^b^	−0.15	−0.28	0.02	0.18^a^	0.18^a^	0.11	0.70	−0.11
Pain	0.64^b^	0.32^b^	−0.20	0.13	0.38^b^	0.42^b^	0.01	−0.01	−0.15
Dyspnea	0.50^b^	0.23^b^	−0.28	0.20^a^	0.17^a^	0.28^b^	0.04	0.03	0.10
Insomnia	0.40^b^	0.26^b^	0.18	0.07	0.22^b^	0.23^b^	0.05	−0.03	0.15
Loss of appetite	0.45^b^	0.21^b^	0.01	0.06	0.34^b^	0.28^b^	−0.01	− 0.04	−0.49^a^
Constipation	0.65^b^	0.33^b^	−0.26	0.13	0.23^b^	0.30^b^	0.09	−0.04	0.17
Diarrhea	0.07	−0.04	−0.56^a^	0.11	0.18^a^	−0.01	−0.04	− 0.11	0.39
Financial difficulties	0.19^a^	0.29^b^	0.03	0.15^a^	0 .20^b^	0.21^b^	0.10	−0.08	−0.12
Global health status/ QoL	−0.64^b^	0.40^b^	0.53^a^	−0.14	−0.33^b^	− 0.35^b^	−0.06	− 0.01	− 0.27

^a^Correlation is significant at the p 0.05 level; ^b^Correlation is significant at the 0.01 level

Known group validity was established by assessing the ability of the EORTC QLQ-CX24 to detect difference in level of HRQoL across patient’s performance status, whether patients being on treatments or not, and FIGO stages. Results differed significantly among patients’ performance status with lymphedema (*p* < 0.05) and the remaining functioning and symptom scales had no significant difference in the HRQoL among the different ECOG-PS. The sexual/vaginal functioning mean score showed a difference among treatment groups. Although not significant, differences in mean score were observed among the groups and scales & items of the EORTC QLQ-CX24.

The study showed that symptom experience, sexual/vaginal functioning scales and sexual activity items were capable of discriminating among patients with different FIGO stages (*p* < 0.05). No significant difference of mean scores for patients with different FIGO stages was found in other functioning and symptom scales of the EORTC QLQ-CX24 (Table 4). For instance, mean scores of the symptom experience, peripheral neuropathy, menopausal symptom and sexual worry of patients with progressive cervical cancer (Stages II-IV) were higher than those with early cervical cancer (Stages I-II). On the other hand, the result for sexual enjoyment and sexual activity showed that as the stage progress, the mean scores become lower, indicating a lower quality of life as well.

**Table 4:** Comparisons between EORTC QLQ-CX24 scores in different FIGO stages, performance status and treatment groups

EORTC QLQ-CX24	ECOG-PS		FIGO stages		Treatment groups	
	Good PS (0–1) (*n* = 145)	Poor PS [2–4] (*n* = 26)	Stage I- II (*n* = 63)	Stage III-IV (*n* = 108)	On treatment (*n* = 83)	Off treatment (*n* = 88)
Symptom experience	42.40 (24.40)	46.15 (19.22)	38.72 (24.20)^a^	45.45 (23.12)^a^	40.67 (25.46)	45.14 (21.78)
Body image	46.89 (39.35)	35.89 (40.28)	45.50 (41.65)	45.06 (38.51)	47.25 (39.02)	43.30 (40.22)
Sexual /vaginal functioning	63.88 (29.56)	75.00 (0.00)	65.27 (29.26)^a^	63.09 (30.37)^a^	54.62 (33.87)	73.33 (21.44)
Lymphedema	10.57 (24.75)^a^	25.64 (39.22)^a^	17.98 (31.00)	9.87 (25.47)	16.86 (32.24)^a^	9.09 (22.4)^a^
Peripheral neuropathy	40.22 (39.06)	42.30 (39.50)	42.32 (38.43)	39.50 (39.50)	44.97 (39.79)	36.36 (38.0)
Menopausal symptoms	53.79 (37.92)	60.25 (35.30)	53.43 (39.04)	55.55 (36.74)	53.81 (37.83)	55.68 (37.38)
Sexual worry	32.41 (46.13)	39.74 (47.15)	39.15 (47.34)	30.24 (45.46)	32.53 (44.77)	34.46 (47.79)
Sexual activity	8.39 (23.55)	2.56 (13.07)^a^	11.82 (26.37)	4.98 (19.31)	8.13 (24.59)	6.89 (20.75)
Sexual enjoyment	37. 03 (32.11)	66.66 (0.00)	33.33 (24.61)	47.61 (42.41)	40.74 (32.39)	36.66 (33.14)

^a^Significantat *p <* 0.05, Independent t-test were used to compare the groups

## Discussion

This is the first study conducted to establish if the Amharic version of EORTC QLQ-CX24 questionnaire is an acceptable, reliable and valid measure to assess HRQoL in Ethiopian cervical cancer patients. The finding of the study revealed that the Amharic version of EORTC QLQ-CX24 has good acceptability and is psychometrically robust tool to measure HRQoL for Ethiopian cervical cancer patients.

All presented Cronbach alpha values exceeded the 0.7 criterion. The internal consistency of the body image scale in this study was found to be consistent with previous studies [9, 18]. Pearson correlation analysis confirmed the satisfactory convergent and discriminant validity of the EORTC QLQ-CX24, as correlations had r ≥ 0.40. Similar to the English version, symptom scale, body image and vaginal functioning have good item convergence [9], which showed that each item was highly correlated with one another as compared to items of another scale. All the items have good discriminant validity except for item 53 of the vaginal functioning scale, which ask about pain during sexual intercourse, having exhibited a higher positive correlation with sexual worry.

Sexual/vaginal functioning and sexual enjoyment were conditional; a patient could skip answering the question if they have not had sexual activity in the past 4 weeks. As a result, 152 (89%) patients skipped the sexual/vaginal functioning scale and sexual enjoyment item. This indicated that the status of the patients highly influenced their sexual activity and functioning. Another studies also indicated that cultural boundaries and privacy of the sexuality could have an influence in HRQoL [11, 19].

The Pearson correlations between the EORTC QLQ-C30 and the EORTC QLQ-CX24 were strong in the majority of the scales. In the Chinese and Polish validation studies [10, 19], the EORTC QLQ-CX24 scores strongly correlated with the EORTC QLQ-C30 core questionnaires, indicating that those scales that assess similar aspects were strongly correlated. Weak or no correlation indicated that the EORTC QLQ-CX24 has unique domains of HRQoL, which are not addressed by the EORTC QLQ-C30. These results are another confirmation on the need to conjugate the cervical cancer specific questionnaire with the general cancer questionnaire in order to assess the HRQoL of a patient with cervical cancer.

The result of the known group validity showed that the EORTC QLQ-CX24 cervical cancer specific module was able to discriminate between patients with different FIGO disease stages and ECOG-PS as well as patients being on treatment or not. The symptom scale differentiates between the early and advanced FIGO stages of cervical cancer.

The lymphedema single item differentiated among the good and poor ECOG-PS of the patients. This is quite different result compared to the English version, which found that symptom experience differentiated among all FIGO stages and treatment options. Other studies shared a similar result with only one sub-scale differentiating between the FIGO stages [10, 19, 20]. The current finding differed from others and such difference might be attributed to differences in the socio-demographics, comorbidities, perceptions about the disease, culture and healthcare service of the hospitals [9]. Only 10% of the patients responded to question about sexual functioning and enjoyment. Since the number of sexually active patients was very small, the significance of the correlation of the scale and items could not be established as well as generalized to the sample size. Furthermore, the response to sexual activity might be attributed to the cultural hindrance regarding sexuality of a woman [9, 20]. In addition, it has been documented that any disease condition of the “sexual area” would influence the patient’s body image and sexual worry regardless of the stages [10, 11].

Finally, this validation study had certain limitations. The study did not measure test–retest reliability and the responsiveness of the EORTC QLQ-CX24. In addition, because of limited number of sexually active patients, the actual correlation of the scales could not be indicated and further research with larger sample size is recommended.

## Conclusions

Amharic version of the EORTC QLQ-CX24 questionnaire is a valid and reliable tool and could be used in clinical and epidemiological cancer researches to study the HRQoL of patients with cervical cancer in Ethiopia.

## Abbreviations

ECOG-PS: European Cooperative Oncology Group Performance status
EORTC QLQ-C30: European Organization for Research and Treatment of Cancer Quality of life Questionnaire version 3
EORTC QLQ-CX24: European Organization for Research and Treatment of Cancer Quality of Life Questionnaire cervical cancer specific module
FIGO: International Federation of Gynaecology and Obstetrics
HRQoL: Health Related Qaulity of life.
TASH: Tikur Anebessa Specialized Hospital.
